# Use of Restorative Justice and Restorative Practices at School: A Systematic Literature Review

**DOI:** 10.3390/ijerph19010096

**Published:** 2021-12-23

**Authors:** Ernesto Lodi, Lucrezia Perrella, Gian Luigi Lepri, Maria Luisa Scarpa, Patrizia Patrizi

**Affiliations:** Department of Humanities and Social Sciences, University of Sassari, 07100 Sassari, Italy; lucrezia.perrella@outlook.it (L.P.); gllepri@uniss.it (G.L.L.); mlscarpa@icloud.com (M.L.S.); patrizi@uniss.it (P.P.)

**Keywords:** school, restorative justice, restorative practices, children, adolescents, literature review

## Abstract

Background: In recent years, the use of restorative justice (RJ) and restorative practices (RP) in schools has grown rapidly. Understanding how theory and research address this topic is important for its practical implementation based on scientific knowledge. The aim of this article was to analyse the practices derived from RJ implemented in school and what kinds of results have been achieved. Starting from the analysis of the qualitative and quantitative research in the field, a systematic review was conducted on the last decade of studies using RJ and RP at every level of school education. Methods: For this review, methods including the PRISMA guidelines, the PRISMA flow diagram, and qualitative synthesis were carried out. Scientific articles for the literature review were selected according to the following criteria: (1) publication date between the years 2010–2021; (2) student population aged 6–18 years; (3) publications in the English language; (4) articles directly accessible or accessible by contacting the author(s); 34 articles met the inclusion criteria. Results: The most used RP in school are circles (*n* = 26), followed by restorative conferences (*n* = 17), peer mediation (*n* = 10), restorative conversations (*n* = 8), mediation (*n* = 7), community-building circles (*n* = 5). RP can improve the school climate, discipline, positive conflict management through actions that aim at preventing suspensions, exclusions, conflicts, and misbehaviours (e.g., bullying). RJ practices promote positive relationships between peers and between students and teachers, as well as to prosocial behaviours through the development of social and emotional skills. Conclusions: From the studies examined, a great interest in applying restorative justice and practices in schools clearly emerged. Discussions on the benefits and challenges of implementation were provided. However, there is still limited evidence in terms of direct correlation, which suggests further studies on the impact of RJ and RP in school settings.

## 1. Introduction

The perspectives of the current international debate, also in the wake of the recent Recommendation CM/Rec (2018) 8, aim at encouraging not only the development and use of restorative justice in criminal matters, but also the development of innovative restorative approaches to be placed outside the justice systems, highlighting how justice and restorative practices do not only concern behaviours of criminal relevance. Indeed, restorative justice and practices may also have a role in the various conflicts arising in different communities (such as schools) not only as a response to conflict, but also as a preventive approach aiming at building relationships and communities. Therefore, it is possible that after harm suffered, there is a need to rebuild the sense of trust and heal conflicts to heal people’s wounds and fractures in the social fabric. The aim is to prevent harmful behaviours towards the expected prospect of a better future: a future of safety, trust, responsibility, and well-being of all the parties involved. In this sense, restorative justice can be presented as a justice for people and relationships, when a crime has been committed, a harm produced, a pain suffered, to prevent harmful behaviour [1].

Current zero-tolerance policies represent systems that use punitive and exclusionary practices (e.g., suspensions) to control and manage student behaviour. These policies very often aggravate disciplinary problems and exacerbate racial, gender, and socioeconomic status disparities, underlining the need for alternative approaches to the management of school discipline, as well as approaches that aim at promoting well-being in the whole school community.

Restorative justice could represent one of these alternatives. Its practices are oriented not only at the alternative management of incorrect and violent behaviours, for example, bullying, but mainly at the promotion of prosocial behaviours through the development of social and emotional skills (e.g., empathy, awareness, and responsibility), with the broader goal of building safe school communities that promote well-being. 

In recent years, the use of restorative justice and restorative practices in schools has grown rapidly. This encourages a better understanding of how theory and research address this question to further improve practices and ensure their application is based on scientific knowledge. Starting from existing qualitative and quantitative research, the aim of this study was the evaluation of the practices implemented and the results obtained through a systematic literature review on the use of RJ and RP implemented at school. We wish to support practices by the existing findings in scientific research, listing the benefits of applying restorative justice and restorative practice in schools and analysing the impact of these approaches in educational settings.

### 1.1. Restorative Justice and Restorative Practices

Restorative justice is preliminarily a paradigm, not identifiable in a specific program [2,3,4,5] or in a specific field of application [6]. Its possible declinations correspond to different programs, which share some key dimensions: (a) a proactive and promotional vision; (b) the offense is not identified with the behaviour, of which it is only a legal definition; (c) the person who carried out the action is a person rather than a judicial role (investigated, accused, convicted); (d) whoever has suffered the consequences is, even before being an offended party or victim, a harmed person. 

This does not mean ignoring the legal significance of the crimes and the people involved (perpetrators and victims), but considering them as persons rather than perpetrators and victims, behaviours that produce harm rather than crimes, consequences rather than victimization, judgment, conviction, punishment [1].

Restorative justice proposes a radically different reading from that of criminal justice. For the latter, crime is a violation of the law and the State, while for restorative justice, crime is a violation of persons and obligations, of harm caused and suffered, of social crises, and this means recognizing people and their actions for what they are. For criminal justice, a violation creates guilt and requires paying with suffering, there must be a punishment. For criminal justice, the focus is on the perpetrator who must pay their debt to justice and to the State who, in this way, completely replaces the victim, giving the latter the role of initiator of the criminal action. For the restorative justice, a violation creates new obligations, through which justice and relational balance can be restored. Restorative justice acts by questioning the assumptions of the judicial system without denying them, emphasizing harm reparation as a means of restoring justice and relational balance rather than punishing incorrect behaviour. Therefore, restorative justice involves those who have suffered the harm, those who are responsible for it, and members of the community in a commitment to make “right” what is wrong, restore justice that is respectful of everyone, of people, and of coexistence [7,8,9,10,11]. Restorative justice focuses on the need of all the involved parties [9]. Victims need to have access to nonjudicial information relating to what has happened: why and what follows the fact that happened (information that often only the perpetrator of the crime possesses); to tell the personal truth on what happened through a space to be heard and tell one’s story; to make sure that those who have acted against them know the consequences they have produced; to regain control over one’s life; to have a compensation. The needs of those who have committed the crime can be to take responsibility for the consequences of their action on the victim and support to progress towards change in view of reintegration into the community. The needs of the community, which must protect its members and itself, are to restore trust in bonds, take care of the person who has suffered, of the person responsible, of all the parties who have an interest in rebalancing and supporting positive relationships. The broadest form of restorative justice is the one that takes place in the encounter and interaction of the three areas of needs.

The paradigm shift represented by restorative justice is evident: crime generates harm and determines needs; justice should work to repair the harm and address these needs. Different conceptions of restorative justice can coexist, but they all share the basic assumptions: encounter, reparation, transformation. These three elements are in agreement with the values of restorative processes and with the needs they are addressing, but each one of them includes aspects that are not necessarily considered by the others: centrality of the encounter and use of restorative processes even in the absence of a crime; centrality of reparation even outside of a restorative process, as in those cases where the victim does not intend to participate; focus on social justice by addressing structural and individual injustices as possible preconditions for crime. 

Within the paradigm of restorative justice, different programs can be devised depending on the vision assumed, the protagonists who take part in it, and the social, economic, and cultural context, as well as the ability to accept alternative conflict management formulas. These programs can be family group conferences; restorative conferences; circles of peace; victim/offender mediation; community-building circles. The possible programs of restorative justice must be, in any case, designed with the awareness that the three protagonists with those three areas of needs must be able to meet and interact within the area of shared principles, even where the specific program does not include all parts.

Such a broad and inclusive vision makes it possible to highlight the fundamental dimensions of the approach since, transversally, they can go beyond the criminal question: restorative justice and restorative practices do not only affect behaviour of criminal relevance, but also conflicts, offenses, and transgressions that can take place in the community and in everyday contexts, and not only as a response to the conflict, but in a preventive approach to care for relationships. In this perspective, restorative justice represents an approach aimed at promoting lifestyles and ability to perform peaceful conflict management oriented towards a social sense of justice in relationships and in communities, trust, inclusion, cohesion, equity, peace, and social support in accordance with the internationally shared values of restorative justice: justice and accountability, solidarity and responsibility, respect for human dignity, search for truth through dialogue (the experience of history for each of the parties involved) [12]. Therefore, the relationships between people represent the main resource for building social bonds, interactions, and opportunities to prevent discomfort and deviance, generate connections of well-being.

### 1.2. Restorative Justice and Restorative Practices at School

Traditionally, school systems use prescriptive and punitive methods to manage, respond, and deal with bad behaviours that students display. These methods, known as zero-tolerance policies, involve actions of exclusion (e.g., suspensions and expulsions) that lead to the removal and isolation of students that commit illegal behaviour from both the school context and the community and that are put in place to enforce order within the schools themselves [13].

The American Psychological Association (APA) Zero Tolerance Task Force [14], analysing zero-tolerance policies, highlighted the negative consequences of these practices: intensification of the inequality of treatment with respect to Black students and urban school students with low socioeconomic status, higher likelihood of recurrence of deviant behaviour, dropout, higher likelihood of crime [14,15,16,17,18]. To address these consequences, it is critical that schools promote and experiment with alternative disciplinary methods, like restorative approaches and practices, to substitute zero-tolerance policies and punitive practices. However, it is essential to think of an approach that is promoted throughout the school, that should be focused not only on repairing harm in the event of conflicts and harmful/violent behaviours (e.g., bullying), but also on building and cultivating relationships, promoting both relational/emotional and peaceful conflict management skills, nonviolent communication, a sense of security, respect, well-being. In this sense, the interventions carried out in the context of restorative justice programs are framed in its promotional perspective, which is a proactive vision that, while acting on the conflict triggered by a harmful behaviour or other illicit actions (e.g., crime), looks at future development of people and their relationships, at their ability to prevent and deal with conflict as the best solution for coexistence. When this approach is applied to contexts such as schools, the aim of promotional prevention is perhaps more immediately visible: some behaviours cause harm, which creates needs, which require restorative responses; restorative responses meet needs, which repair harm; repairing the harm may or may not lead to the prevention of crime and/or harmful behaviour [19]. Indeed, many programs developed in schools “can provide an opportunity for the community to provide an appropriate educational response to minor offences and other conflicts without formally criminalizing the behaviour or the individual” [20].

**Figure 1:**
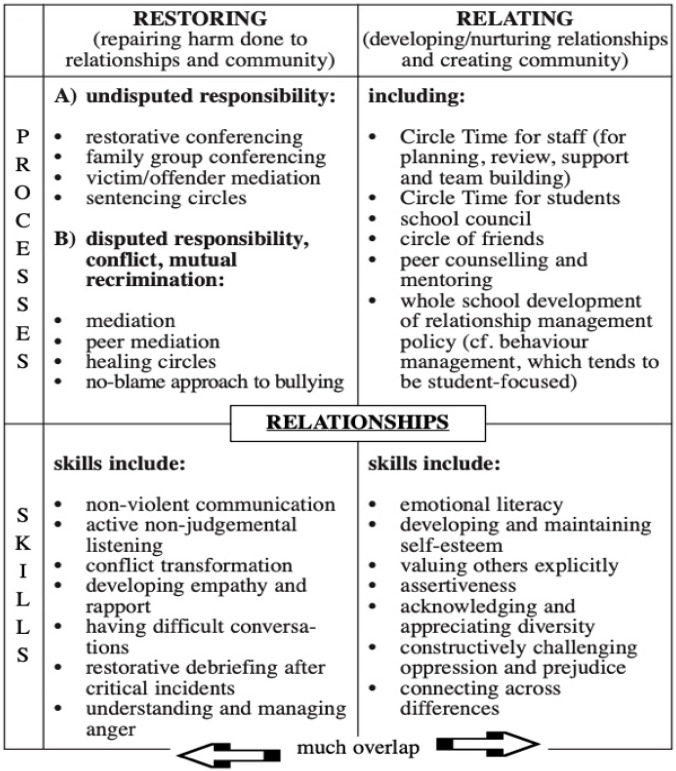
Restorative and relational process skills [21]. Reprinted with permission from Ref. [21] Copyright 2021 Belinda Hopkins.

In the international context, in recent years, more schools have launched and tested initiatives and projects aimed at promoting the restorative approach and restorative practices: (a) in terms of cultural and disciplinary policy of the entire school, supporting students, teachers, non-teaching staff with specific training; (b) as an approach capable of promoting and developing social and emotional skills (e.g., empathy, self-esteem, nonviolent communication, peaceful conflict management); (c) as practices specifically activated, with the involvement of external facilitators, to manage and respond to episodes of bullying, conflicts, inappropriate and/or offensive and/or violent behaviour. Figure 1 is an example of this. Therefore, involvement of the school community in the resolution of conflicts that may arise within the same is based on the idea that members of the community need and want to repair the harm suffered and/or acted upon and that they have the skills and opportunities to do it, promoting the development of creative resolution strategies, nonviolent communication, and non-judgmental listening. The starting point is that the promotion of the restorative approach to the whole school, through the activation of practices such as peer mediation, circle time, restorative conferencing, family group conferencing, community-building circles, can represent an approach aimed not only at repairing harm in case of conflicts and/or incorrect behaviour, but which allows building and strengthening relationships, as well as promoting and developing relational and personal skills such as empathy, assertiveness, self-efficacy.

## 2. Materials and Methods

For the purposes of the systematic review of the literature, those studies that activated restorative justice interventions at school were taken into consideration. By interventions we mean activation of restorative practices, such as circles, conferences, mediation, peer mediation, restorative conversations, community-building circles, conducted by:

(a) external experts, such as facilitators and/or a mediator;
(b) teachers and students trained on the use of restorative practices;
(c) researchers as experts in restorative practices.

A systematic review of the literature was conducted following the PRISMA guidelines (Preferred Reporting Items for Systematic Reviews and Meta-Analyses) and the PRISMA flow diagram (http://www.prisma-statement.org accessed on 8 March 2021) adapted to the type of synthesis proposed by this review. Indeed, a qualitative summary and not a meta-analysis summary was performed in this review.

A systematic literature search was conducted between March 2021 and June 2021. To answer the research questions, only articles that met these inclusion criteria were selected:

- Documents: scientific articles published in the selected databases;
- Population: age of 6–18 years, schools, males, females;
- Interventions: restorative justice and restorative practices;
- Sources of information: databases (Web of Science, Science Direct, PubMed, APA PsycInfo, APA PsycArticles, Psychology & Behavioural Sciences Collection, Education Research Complete);
- Type of publications: full-text papers already published or accessible on request through contact with the authors;
- Language: English;
- Years of publication: 2010–2021.

Consequently, documents such as conference proceedings, books, book reviews, and dissertations were excluded. 

**Figure 2:**
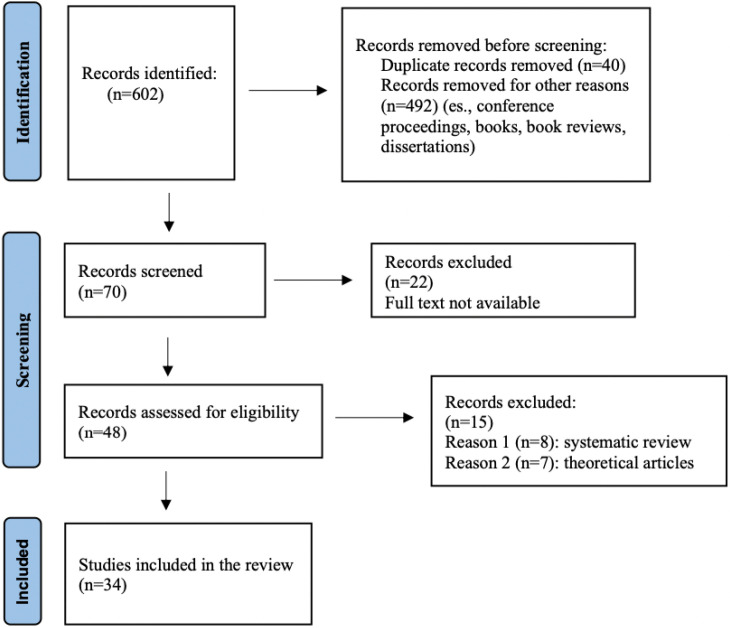
PRISMA flow diagram of the study selection process.

The research was performed in the different selected databases (Web of Science, Science Direct, PubMed, APA PsycInfo, APA PsycArticles, Psychology & Behavioural Sciences Collection, Education Research Complete), limiting the search to the publications published in the years 2010–2021; 602 articles were found based on the search carried out with the keywords “school” and/or “restorative justice” and/or “restorative practices” and/or “children” and/or “adolescents”. Figure 2 presents a PRISMA flow diagram of the article’s selection process.

For the scoring process, the PRISMA Checklist was used for the items to be included in the reporting of a systematic review. All the records were downloaded and inserted in an Excel file, indicating for each full-text availability/non-availability. The full-text articles were downloaded while the unavailable articles were requested from the authors (*n* = 22).

All the 602 articles were subjected to the initial manual selection (title and abstract) with the involvement of two professional figures. The articles were further examined by two other professional figures: out of the 602 articles found, 40 duplicates were removed, and 492 were excluded (conference proceedings, books, book reviews, doctoral theses). There were 70 records evaluated for admissibility; of these, 21 records were excluded because full texts were not available: none of the requested articles were sent.

Additional 15 articles were excluded from the final analysis as they are systematic reviews and theoretical articles. As academic scientific literature on the topics covered by this review, these 15 articles are reported in Appendix A (Table A1) as a qualitative summary as a possible object of interest to the reader.

There were two articles which the research team did not agree to include in the review, therefore an opinion was sought from an external expert with international experience on restorative justice: their answer was discussed by the entire research team, and finally two articles were included in the review.

Thirty-four articles were assessed as eligible and therefore included in the qualitative summary. A codebook was created for the 34 selected articles using Microsoft Excel v16.56 (21121100) and the IBM SPSS Statistics for Windows, Version 25.0. (IBM Corporation, Armonk, NY, USA) for graphs and percentages. The first version of the module was created of the selected articles (50%) and then verified (the remaining 50%) by two independent coders [22]. Some categories of results were modified, separating and/or summarizing them (e.g., risky behaviours for health; promotion of well-being; interpersonal relationship improvement; disparities; safety; school climate; academic results; absenteeism).

The two independent experts coded the articles according to the constructed categories. Only in five articles, there were different modes of interpretation by independent coders (only one category interpreted differently by coders for each article). The five articles were submitted to two other independent coders and subsequently to the research supervisor. The final discussion with the supervisor and research team resolved the doubts on these five articles.

## 3. Results

Thirty-four studies conducted in seven countries (USA, 23; UK, 4; Australia, 2; Canada, 2; Croatia, 1; Japan, 1; Scotland, 1) were included in this systematic review. Of these, six studies are randomized controlled trials (RCT) [23,24,25,26,27,28]; one study is a follow-up survey [29]; one study is a correlational study [30]; one study is an interrupted time series (ITS) analysis [31]; two studies are nonexperimental design studies [32,33]; one study is quasi-experimental pre–post design study [34]; 17 studies are qualitative studies (in these, we considered single-case studies) [35,36,37,38,39,40,41,42,43,44,45,46,47,48,49,50,51]; one study is both literature review and a qualitative research study [52]; two studies are qualitative and quantitative studies [53,54]; two studies are quantitative studies [55,56].

The characteristics of the 34 included studies and the qualitative synthesis are reported in Appendix B (Table A2) following the PICOS scheme: participants, interventions, comparisons, outcomes, and study design.

Twenty-six studies [24,25,26,27,28,30,31,32,33,34,35,36,37,38,39,41,42,43,44,45,48,49,50,51,52,54] within the projects for the implementation of restorative justice and restorative practices at school provided for the activation of training courses in restorative justice and the use of its practices. The training of teachers, school staff, and students has made it possible to sensitize the entire school to the restorative approach; supporting openness to change in school policy and facilitating the application of the restorative approach to the whole school; transferring knowledge and skills; making students and teachers autonomous in the activation and management of restorative practices; develop skills to manage and deal with conflicts independently; enable students to become active members of school life and decision-making processes on issues that concern them. Of these 26 studies, 20 studies [24,25,26,27,28,31,32,33,35,36,37,38,39,41,44,45,48,51,52,54] envisaged the involvement of external experts both to support students and/or teachers in the management of the practices and because they were called to be their facilitators. In eight studies [23,29,40,46,47,53,55,56], the RP were provided directly by the researchers as experts in restorative practices. 

The people involved in the studies as the participants were students, teachers, principals, non-teaching staff, parents, for a total 22,383 participants from about 900 schools.

The definition of the school level involved in RP intervention is not a consistent concept across different studies with different jurisdictions. However, we summarised the school level in the following categories: primary school/elementary school (14.7%); middle school/secondary school (20.6%); high school (17.6%); elementary/primary, middle/secondary and high school (5.9); middle/secondary and high school (17.6%); primary/elementary school and middle/secondary school (17.6%); not available (5.9%). This result seems consistent with the age range of the students involved: 6–11 (11.8%), from 9–12 to 11–14 (35.3%), 14–18 (17.6%), 11–18 (17.6%), 5–18 (11.8%), not available (5.9%).

The studies included in this review dealt with analysing the impact at the individual and/or school level of the implementation of restorative justice and restorative practices, both as a whole school-oriented approach and as practices activated to respond to specific cases through comparison with the period prior to implementation or with schools that promote traditional and/or punitive school disciplinary policies (e.g., zero-tolerance policies); in particular, in 47.1% of the studies, the schools envisaged traditional disciplinary practices; in 20.6% of the studies, the schools had zero-tolerance policies; in 20.6% of the studies, the schools envisaged traditional disciplinary practices oriented towards punitive and exclusive approaches; in one study (2.9%), the comparison was made with the nonapplication of action groups formed in restorative justice; in 8.8% of the cases, it was not possible to identify the disciplinary approach prior to the study.

### 3.1. Restorative Practices Used in School

In all the 34 articles examined, it emerged that each school had activated and implemented at least one restorative practice both as daily educational practices that teachers can use in classrooms and as practices to manage, deal with, and respond to minor and/or moderate and/or serious gravity; harmful behaviours; violence; school crimes.

**Figure 3:**
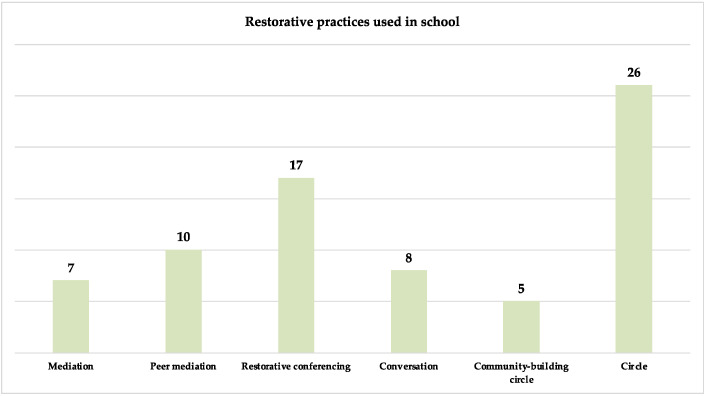
Restorative practices used in school.

As you can see in Figure 3, the most used RP was circles (*n* = 26), followed by restorative conferences (*n* = 17), peer mediation (*n* = 10), restorative conversations (*n* = 8), mediation (*n* = 7), community-building circles (*n* = 5). 

These practices, inserted within a project to implement the restorative approach across the whole school, can become practices inserted within the didactic school curriculum as they can lead to an increase in students’ social skills (e.g., empathy, awareness, responsibility), foster ability to express and manage emotions, promote the development of fair and positive relationships. For example, several schools implemented community-building circles in classrooms. Therefore, community-building circles represent preventive methods that can be designed and activated to support students and teachers in developing strong and positive relationships between them: these circles allow creating a safe relational space in which students can tell their own stories of life and experiences, thus encouraging learning and mutual knowledge. Furthermore, in some schools, it is sometimes the students themselves who lead the circles rather than the teachers.

Restorative conversations were activated to discuss general life issues (e.g., politics, sports, etc.), issues concerning teaching and/or school in general, but also as moments of listening and preparation for students in view of a restorative process.

It emerged that many schools activated mediation with the support and guidance of qualified external staff (e.g., facilitators as a neutral third party) to address mostly serious conflicts between students. Peer mediation and restorative circles were activated to manage and respond to minor conflicts. With respect to peer mediation, the literature underlines the importance of training students in this practice as it could represent a beneficial process for the whole school: through peer mediation, students can experiment using their conflict resolution skills, become able to independently manage problems, conflicts, differences, repair and build relationships, and feel being an active part of the decision-making process by building shared solutions themselves and not letting the school solve problems for them.

Restorative circles and conferences can represent alternative approaches to managing student behaviour problems, creating a space for reflection and discussion to find alternative disciplinary responses to suspensions and exclusions. As with mediation, restorative conferences are mostly implemented as a response to serious conflicts, while restorative circles are mostly implemented in response to minor conflicts. Both practices, very often facilitated by external personnel, allow building listening moments and spaces in which to co-construct the assumption of responsibility, actions, constructive responses. Circles are mainly activated to address specific problems (e.g., racism and bullying in the classroom), while conferences—to address the most serious incidents (e.g., school crimes, violence) involving all the parties involved and/or affected by the harmful behaviour: the participants always include the victim, the perpetrator, and the facilitator, but also other members of the school community, such as students, families and, if and/or when necessary, external agencies as well.

Harm and support circles are activated to manage and respond to conflicts and/or other issues (e.g., absenteeism), involving both the interested parties and the key support stakeholders. Harm and support circles are also generally run by trained staff, most of the time—by a facilitator.

Some schools, in parallel or within an RJ intervention, planned to include individual and group counselling moments. Sometimes, counselling represented a moment of tutoring between the school staff and the student, including in terms of activating a process to support students for school reintegration following a disciplinary measure of suspension or exclusion from the class.

### 3.2. Effects of the Restorative Approach and Restorative Practices

Positive results emerged with respect to different aspects: school climate, discipline, positive conflict management through actions that aim at preventing suspensions, exclusions, conflicts, and misbehaviour (e.g., bullying); positive relationships between peers and between students and teachers; prosocial behaviours; social and emotional skills; school–community–family ties; well-being (through restorative culture as a whole-school approach).

**Figure 4:**
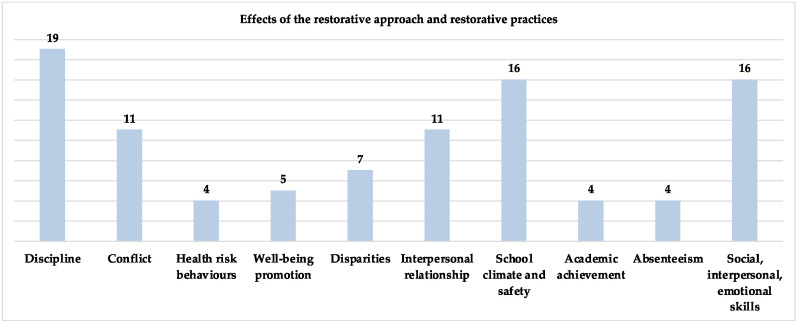
Effects of the restorative approach and restorative practices.

The effects of the restorative approach and restorative practices most reported in the studies concern, as you can see in the Figure 4, discipline (19 studies); school climate and safety (*n* = 17); social, interpersonal, emotional skills (*n* = 16). The other results reported by the studies concern conflict (*n* = 11), interpersonal relationship (*n* = 16); disparities (*n* = 8), well-being promotion (*n* = 5); health risk behaviours (*n* = 4); academic achievement (*n* = 4), absenteeism (*n* = 4).

#### 3.2.1. Discipline and Disciplinary Sanctions, Bullying, Violence, Unequal Treatment 

Nineteen studies highlighted how participation in restorative interventions and/or programs can bring positive results in terms of greater ability to manage behavioural problems and school discipline, with greater adherence to the rules. In schools where the restorative approach (and practices) is adopted, less misconduct by students, reduction in injuries, disciplinary postponements, and school crimes are evident, with more positive behaviours and decreases in suspension rates and disciplinary sanctions. This leads to a reduction in the need for punitive measures [24,25,26,27,28,30,31,34,36,38,39,40,41,42,46,48,50,54,55]. For example, the study by Payne et al. [55] pointed out that the use of severe punishment increases the likelihood of choosing punitive/zero-tolerance policies to manage and respond to student behaviour problems; the use of milder punishments increases the likelihood of resorting to restorative practices; the use of harsh punishments is unrelated to the level of crime and delinquency. According to four of these 19 studies mentioned, a whole school-oriented restorative approach leads to the development of alternative methods to punitive and exclusionary discipline and/or sometimes using both punitive and restorative disciplinary responses, also showing a reduction in the use of disciplinary sanctions, such as suspensions and expulsions and/or fairer disciplinary practices [27,34,38,39].

Some restorative justice programs and interventions are activated in school settings to prevent, manage, and contain bullying and/or other violence. Eight studies highlighted the reduction in the experiences of aggression, violence, and bullying in the schools that had adopted the restorative approach and restorative practices compared to the schools that used the traditional disciplinary systems and/or zero-tolerance policies [24,25,26,27,28,34,41,42]. There was a reduction in bullying-related behaviours and more reports. In this respect, the collaboration of the principals and the entire teaching staff with the students and families in the management and resolution of such behaviours is fundamental [34]. Four studies highlighted that the use of restorative practices helps to reduce victimization of bullying and cyberbullying [23,24,25,26]. Fewer cases of violent behaviour, aggression, violence, and bullying, fewer victimization situations, and, more generally, fewer school crimes lead to a reduction in costs for society compared, for example, to health services and costs related to the justice system [24,25,26].

Five studies highlighted how zero-tolerance policies exacerbate inequalities in disciplinary treatment between White students and students of a different ethnicity, class, gender, and socioeconomic status; on the contrary, the restorative approach can favour the reduction in the inequality of treatment [27,30,31,38,40]. Anyon et al. [30], while highlighting the positive results of the implementation of restorative practices on disparity, underlined the importance of investing additional resources to ensure that the restorative approach is increasingly promoted in schools, especially oriented throughout the whole school, as there is always a tendency towards unequal treatment in school discipline. Gregory et al. [38] reported that Black students are more likely to be punished, e.g., with suspensions and exclusions, for early misconduct than White students. According to the analysis by Hashim et al. [31], programs of restorative assistance are shown to have considerable suspension rates of students, without rates (Blacks, Latinos, Asians, Whites, low socioeconomic status).

#### 3.2.2. Conflict Management

The restorative approach applied to the school context can be effective in the face of various types of difficulties, including conflicts (between students, between students and teachers, between teachers and parents, between managers and teachers, etc.). The restorative approach and practices represent a working methodology for the resolution of the different conflicts that may emerge as well.

Indeed, 11 studies show that the application of restorative practices and, more generally, of the restorative approach to the whole school, favours the development of alternative practices, strategies, and methods of conflict management and resolution [27,29,31,37,38,41,45,46,48,52,53]. Therefore, according to these studies, an increased proactive conflict management capability emerges, a high level of success of restorative practices in conflict resolution, development of nonviolent conflict resolution strategies, nonviolent problem-solving strategies, and development of skills for peaceful conflict resolution. In these programs, it is very often school staff, sometimes even with the help of external facilitators, who engage students in using restorative practices to resolve, manage, and respond to peer members without using punitive practices; re-establish the rules violated by the behaviour; repair harm; encourage the re-entry into the school for students who have been suspended and/or expelled; teaching students to resolve their conflicts on their own in a peaceful way; promote the ability to identify needs and actions useful to repair the relationship and/or harm between all the parties involved. Among these 11 studies, only from the study of A. Gregory [38] it emerged that restorative practices are also used in schools to resolve and manage conflicts between school staff members.

As pointed out by Peurača et al. [52], sometimes, conflicts are an integral part of community life, such as school, and can represent valid opportunities for growth, but very often the inability to manage and face them or manage them and deal with them with punitive practices, leads to opposite effect. On the other hand, it is fundamental to promote alternative and nonviolent methods that can favour the possibility for people to confront each other with respect to different opinions, beliefs, and values as well as have greater awareness of the situation and work together to find solutions. Restorative processes can be a nonviolent response: they help to prevent and reduce conflicts and resolve them peacefully.

González et al. [37] and K.E. Reimer [46] explored the association between restorative justice and student well-being, and as regards the perception of students about conflicts, the restorative approach implemented at school allowed students to reread conflict as a normal and natural aspect in relationships if it is addressed and managed as a moment of mutual growth. Therefore, students reported that conflict management-oriented restorative practices allowed the school community to work together in a proactive, constructive, and respectful way, exposing various problems and finding together strategies and solutions to solve them: the restorative approach and practices can promote participatory and cocreative decision-making processes between students and school staff.

Of the 11 studies, five studies found that student engagement by teachers in conflict resolution or respect issues that deal with conflicting topics and/or experiences promotes dialogue and reflection on how to resolve them in a different and peaceful way, bringing out a greater capacity for proactive management. The importance of promoting proactive practices that are used in everyday life, both in the classroom and at school, is highlighted to foster positive relationships and prevent the onset of conflicts [27,45,46,48,53]. Furthermore, Sandwick et al. [48] spoke of the restorative approach in terms of a “holistic framework” since, precisely because of its transversality, it does not promote the building of relationships and the resolution of conflicts only when disciplinary incidents occur but is perfectly integrated into the functioning of the school.

#### 3.2.3. Health Risk Behaviours

Four studies [24,25,26,28] highlighted the effects of restorative justice and restorative practices on health risk. It emerged that the use of the restorative approach at school reduces the likelihood of students engaging in harmful behaviours for health such as the use of substances, alcohol and drugs, smoking, and dangerous sexual intercourse. For example, the three studies by Bonell et al. [24,25,26] showed that restorative interventions had a greater effect in boys than in girls with respect to the quality of life, psychological problems, well-being, smoking and alcohol, bullying, contacts with the police. Furthermore, the proposed interventions were more effective with students who had had different bullying experiences, significant effects on psychological difficulties, quality of life, and well-being, and with students with greater aggression, with significant effects on the quality of life, psychosocial problems, well-being, and some risk behaviours (smoking, alcohol). Even Warren et al. [28] showed greater efficacy of intervention in males than in females with respect to the reduction in health risk behaviours, such as smoking, drugs, alcohol, fewer contacts with the police for risky behaviours, and with the health system, in their study. Some studies tried to analyse the possible relationship between the use of the restorative approach at school and the improvement of well-being. 

#### 3.2.4. Well-Being

Five studies [28,33,42,46,56] investigated the impact of restorative justice and restorative practices on well-being. The research by Norris [33] focused on evaluating the impact of the restorative approach on two areas of psychological well-being, happiness, and school commitment. The authors stressed, on the one hand, the potential of the restorative approach in positively influencing the outcomes, finding higher levels of happiness and scholastic commitment in schools that have implemented the restorative approach to the whole school; on the other hand, the author highlighted the lack of empirical evidence to support this thesis, especially with respect to the positive influence of restorative programs on happiness: it is not clear which restorative practices are responsible for this positive influence. Reimer [46] suggests that the restorative approach and practices, aiming to build solid, positive, lasting, and quality relationships, helped students to give and understand the meaning of their daily existence, generating and promoting feelings and perceptions of individual and collective well-being. Even Warren et al. [28] showed positive outcomes of the restorative justice approach in greater mental well-being, better psychological functioning, and quality of life, with an increase in health and well-being throughout the school in their study. Furthermore, from the study by Todic et al. [56], it appears that in schools that have implemented restorative justice, students are less likely to be absent from school due to health problems and, according to Kehoe et al. [42], there is also a decrease in the rates of anxiety and depression.

#### 3.2.5. Interpersonal Relationships

Sixteen studies showed how the restorative approach can promote positive interactions between and with all its components. A better student experience both in the classroom and at school linked to improved relationships between peers, between students and teachers, referring to the students’ experience of their teachers as respectful, and between school and families, building mutual trust and growth, emerged [23,24,25,26,28,29,37,38,39,41,42,43,48,50,52,53]. Ingraham et al. [41] stressed the importance of using active, collaborative, and relationship-oriented methods of participation that involve teachers, parents, and students. The two studies by Gregory et al. [38,39] highlighted a greater positive relationship between students and teachers following the high level of implementation of restorative practices by teachers, also affecting the relationship with students of different ethnicities, lowering the level of inequality and promoting equity and social justice, as well as raising the level of perception of teachers as more respectful. This led to reduce the level of unequal disciplinary treatment in terms of fewer postponements and exclusions. The study by Sandwick et al. [48] highlighted that the implementation of the restorative justice approach and restorative practices enable students and parents to consider school staff (teaching and non-teaching) available, supportive, and attentive. The key method for building the relationship between students and teachers was to promote moments of confrontation and personal support and study between them, allowing a flexible and informal development space and favouring opportunities for questions, even the ones far from academic. Another key method highlighted was mentoring for students by all stakeholders who are in contact with the students (teachers, guidance counsellors, social workers). This has made it possible to create and cultivate solid positive relationships, greater trust, and more familiarity, thus facilitating communication and communication of possible problems and conflicts. 

#### 3.2.6. Disparities

Eight studies [27,37,38,40,41,44,47,50] highlighted how restorative practices and even more the restorative approach to the whole school, by building positive interpersonal relationships, can contribute to lowering the level of racial, cultural, gender, and socioeconomic status inequality and exclusion, facilitating positive relationships between students and between students and teachers regardless of the “diversity of the other”. Therefore, from these studies, it emerged that implementation of the restorative approach to the whole school promotes school connection by supporting the development of fair, solid, and trusting relationships and development of the ability to recognize the experience and reality of marginalized student groups: this represents an important protective factor for the most vulnerable, rejected, excluded, marginalized students. In addition, it emerged that RJ interventions can improve relations between the Whites and the Blacks and, consequently, represent an important tool for addressing racial disparity through the development of reflexivity and critical conversations about race as, according to the authors, the Whites and the Blacks differ in the perception of racial discrimination.

#### 3.2.7. School Climate and Safety

The restorative approach is one of the tools that can be considered and used by school staff to encourage the development and promotion of a positive school climate. Indeed, in 17 studies, significant changes in school climate and school safety emerged in schools that promoted the restorative approach. These studies found that in schools that promote restorative practices and, even more so, restorative justice as a school policy, there is a perception by teachers and students of a better school climate and an equitable environment, safe, supportive, inclusive and, more generally, of an improved school environment [23,24,25,26,27,32,33,34,37,38,40,41,42,43,45,46,48]. Of these, only one study [48] showed an increase in the sense of academic value and community support. The study by Reimer [46], exploring the role of restorative justice in facilitating student well-being, highlighted that restorative justice policies and practices implemented in schools had supported and facilitated students to build a strong sense of individual and collective coherence within the school. Therefore, the author stressed that the restorative practices implemented in the school made it possible to create a place where students could “connect with each other”, facilitating and promoting creation of a strong sense of school community. In this sense, the students felt like active members of the community, especially with respect to the decision-making processes of the school.

According to Acosta et al. [23], implementation of the restorative approach, while not causing significant improvements in the school climate, results in the students who have had the opportunity to experience the restorative practices implemented by the school/teachers reporting a significantly higher degree of school connection compared to when these practices are not implemented. 

González et al. [37] found that implementation of the restorative approach to the whole school helps to develop non-hierarchical leadership and promote proactive and co-creative decision-making processes between all school members, strengthening and promoting a strong sense of membership. Ingraham et al. [41], promoting the restorative justice approach and practices as a disciplinary model in school, underlined the importance of using methods and practices of multicultural interviews and collaboration to foster a positive school climate, but also to promote the restorative approach within domestic contexts, contributing to promoting a sense of belonging and a culture of care. 

One other study, not included in the previous ones, in particular, the study by Farr et al. [35] highlighted that to ensure creation of a positive and safe school climate through the restorative approach, the following is essential: openness and readiness for change of the whole school body; collegial support among the staff; strong, shared, and non-hierarchical leadership; work together with teachers and families, strengthening the school–community–family ties. 

#### 3.2.8. Academic Outcomes and Absenteeism

Four studies [28,37,50,56] showed positive results in schools that had implemented the restorative approach and practices with respect to academic outcomes. Consequently, it emerged that higher school performance can contribute to lowering levels of school absenteeism [28,37,50,56]. Restorative practices as an alternative school disciplinary model can also lead to positive results with respect to academic outcomes with higher student engagement in education, supporting educational approaches to improve school performance, and absenteeism at school. Indeed, the studies showed improvements in academic achievement and lower levels of student absenteeism, although there is still limited and conflicting evidence. According to Weaver et al. [50], the increase in academic achievement in classes that implement restorative justice could be because in those classes, the teachers, through restorative justice, are able to create an equitable, safe, inclusive classroom climate which allows, consequently, the students to express themselves, engage, and confront each other and the teachers themselves. 

#### 3.2.9. Social, Interpersonal, and Emotional Skills

Sixteen studies highlighted that the restorative approach and restorative practices can also have positive results in the promotion and development of assertive skills, problem solving, emotional awareness, prosocial behaviours, and, more generally, of social and interpersonal skills. The use of restorative practices in schools promotes the construction of empathy as students can express their emotions, listen, and understand the emotions of others, reflect on their feelings, thoughts, and actions, both past and future, developing such skills as reflective thinking and the ability to take responsibility for one’s own behaviour. Therefore, these studies highlighted how this approach can favour the development of social, interpersonal, and emotional skills (e.g., self-efficacy, empathy, problem solving, awareness, and accountability), perspective-taking, self-awareness, communication skills through participation in dialogue [23,24,25,26,29,34,37,41,42,43,44,45,50,51,52,53]. Furthermore, from the study by Ahmed et al. [29], it emerged that restorative justice and practices, when applied to bullying situations, can lead to the development of shame management skills.

Bonell et al. [24,25,26] promoted teaching training on restorative practices, relationships, and social and emotional skills. It emerged that participation in these trainings led to an improvement of social, interpersonal, and emotional skills (e.g., self-efficacy, empathy, awareness, assertiveness, accountability). These trainings, divided into modules, were then provided to a school so that it could continue the work itself and include these modules within the school program, given the positive results that emerged. The study by González et al. [37] found that the whole-school restorative approach creates opportunities to increase communication and develop and improve human agency and resilience, socioemotional listening, leadership and professional skills. From the study by Ingraham et al. [41], it emerged that the restorative justice approach and practices as a disciplinary model promoted in school favour the learning of listening skills, communication, empathy, and assertive skills, underlining how this learning can also bring benefits in other contexts and in relationships with other people, such as teachers and parents. Wong et al. [34] and Kehoe et al. [42], examining the impact of restorative practices at school, found that the implementation of and experimentation with restorative justice allowed the development of social skills such as harmony, empathy, awareness and responsibility, respect-oriented relationships, reflective thinking. Finally, from the study by Parker et al. [45], it emerged that teachers’ implementation of dialogue-oriented restorative practices enabled them to teach skills for active listening, empathy, perspective-taking, and self-awareness.

## 4. Discussion

The purpose of this article was to analyse which restorative justice practices have been implemented at school and what kind of results have been achieved, starting from the analysis of the qualitative and quantitative research in the field. It emerged that the most used restorative practices concern the involvement of more people (circles and restorative conferences) rather than simply involvement of the victim and the perpetrator of a harmful behaviour, confirming a broader, more systemic, and relational approach to restorative justice. Some schools have promoted the implementation of restorative justice as a whole school-oriented approach in order to change the school and the disciplinary policy that had characterized the schools up to that time, such as traditional approaches and zero-tolerance policies. In this sense, several reflections on punitive and exclusionary school disciplinary policies have emerged, defining them as a matter of health justice and underlining the importance of implementing alternative disciplinary practices, such as restorative justice practices. Traditional approaches and/or zero-tolerance policies very often exaggerate the inequalities of treatment of students of different races, gender, socioeconomic status and increase the likelihood of recurrence of deviant behaviours and criminal behaviours, as well as school dropout. These modalities involve actions of exclusion that lead to stigmatization even more, distancing, and isolation of young people from the school context and from people in general, increasing fragility and vulnerability even more.

Very often, some schools have activated restorative justice practices, including via involvement of external experts, to respond to cases of serious conflict and violence, as well as in cases of bullying, as restorative practices promote active involvement in the processes of solving a bully/victim problem [57]. Sometimes, conflicts are an integral part of community life, such as school, and can represent valid opportunities for growth, but very often the inability to manage and face them or manage them and deal with them with punitive practices leads to an opposite effect. However, it is fundamental to promote alternative and nonviolent methods that can favour the possibility for people to confront each other with respect to different opinions, beliefs, and values as well as have greater awareness of the situation and work together to find solutions. Restorative processes can be a nonviolent response: they help to prevent and reduce conflicts and resolve them peacefully [58]. The potential of interventions and/or restorative programs was highlighted with respect to a greater ability to manage and respond to behavioural problems. Therefore, schools that implement the restorative approach and practices achieve improvements in school discipline, reduction in injuries, disciplinary postponements, and school offenses. Consequently, there are greater positive behaviours and lower suspension rates and disciplinary sanctions, less need for punitive measures. Indeed, interventions and restorative justice projects at the whole school lead to the promotion of alternative and multilevel methods of managing behavioural problems [59].

In line with the scientific literature on the subject, restorative practices not only represent alternative practices to managing and responding to incorrect and violent behaviours (e.g., bullying and school crimes), but also significant spaces and opportunities to tell one’s opinions and emotions, lower the level of disciplinary disparity between students of different races, cultures, and gender, proactively participate in decision-making processes. In addition, some studies showed better academic performance, resulting in lower levels of absenteeism. In this sense, in line with the academic scientific literature, in schools that have implemented restorative justice and restorative practices, there is a slight increase in the average grade, an increase in graduation rates, and a more than double decrease in dropout rates [60]. Therefore, the implementation of the restorative approach and restorative practices is expressed not only as a response to conflict, but in a preventive key to welcome and care for people, relationships, communities.

Furthermore, the restorative approach allows promoting prosocial behaviours through the development of social and emotional skills (e.g., responsibility), listening skills, and peaceful conflict resolution, positive interpersonal relationships, and trust, greater collaboration between school, police, justice system, families. This approach, by changing the entire school environment, could be one of the most effective and efficient ways to build safe, equitable, and inclusive school communities that promote empowerment, well-being, and better quality of life of all members. In addition, the restorative approach can contribute to a significant reduction in school exclusion and inequalities of gender, race, and socioeconomic status [61]. In this perspective, a more general focus can be found in the use of the restorative approach for the management and promotion of interpersonal relationships: positive relationships in the school context (with parents, teachers, peers) are associated with positive outcomes in many spheres of children’s and adolescents’ individual and educational development, affecting school engagement, achievement, and well-being [62]. Scientific research on youth well-being adopts perspectives that aim at improving the quality of life of people, with a specific focus on those protective factors at an individual and contextual level (for example, positive school adaptation) that can promote well-being and/or favour factors and behaviours harmful to health [63,64]. It is also a social level issue “since understanding adolescents’ needs related to mental health is a basilar issue to let young people not only to fulfil their potential but also to contribute to the development of our communities” [63] (p. 125). For this reason, the effects of restorative justice and restorative practices on the management and containment of health risk behaviours should also be emphasized. Therefore, it was found that the use of the restorative approach at school reduces the likelihood of students engaging in harmful behaviours for health such as the use of substances, such as alcohol and drugs, smoking, and dangerous sexual relations.

The possibility of activating training courses for RJ and its practices represents an important opportunity to obtain long-term benefits as it allows sensitizing the entire school to the restorative approach, supporting the openness to change of the school policy, transferring knowledge and skills, making students and teachers independent in the activation and management of restorative practices. In fact, in 26 schools, within the implementation projects of restorative justice and restorative practices at school, awareness-raising training courses were provided for students, teachers, school staff and specific in-depth courses for some staff members for timely conflict management. In 20 of these 26 studies, the involvement of external RJ professionals, such as facilitators, psychologists, social workers, emerged both for the intervention in cases of bullying, conflicts of medium/serious gravity, other harmful behaviours, and to support teachers and/or students in the activation and management of restorative practices. Sometimes, RP were provided directly by the researchers as experts in restorative practices (*n* = 8).

Training students in restorative practices allows them to develop skills to manage and deal with conflicts independently, making them active members of school life and decision-making processes on issues that affect them and makes it possible to promote and develop openness to change in school policies, thus facilitating the application of the restorative approach to the whole school. Conversely, schools that did not provide specific training encountered many difficulties in implementing the restorative approach within the school due to a lack of knowledge of the approach and practices. This underlines the importance of adequate training and support to help teachers, principals, students to gain confidence and become capable in using RJ and its practices and implement the restorative approach as a school policy. 

In addition, complexity has emerged in implementation of the restorative approach and restorative practices at school. First, not all schools are ready and willing to change disciplinary and school policies, and not all schools believe this is possible. In addition, the complexity of implementing restorative justice may be underestimated: it is essential to carry out a careful assessment of the needs and contextual characteristics of the school/community in advance to assess the effective possibility of implementing restorative justice and practices. Although it is essential to establish qualitative models and evaluation systems with respect to the application of these practices, it is not always possible (if ever possible) to apply a standard and unambiguous model. Difficulties have emerged in implementing restorative justice as an alternative disciplinary strategy if one chooses to integrate it into the school without taking action to eliminate one system over the other. Furthermore, it is essential to customize the programs and procedures through a context analysis that identifies the strengths and possible areas to be exploited, starting with small changes up to extending the intervention to the whole school through an action plan shared by the whole school community.

From the studies examined, results of great interest emerged regarding the benefits of applying restorative justice and practices in schools, as also confirmed by the theoretical articles and reviews reported in the Appendix A [57,58,59,60,61,65,66,67,68,69,70,71,72,73,74]. However, there is still limited evidence in terms of direct correlation, which suggests further studies.

## 5. Conclusions

This review was conducted following the Preferred Reporting Items for Systematic Reviews and Meta-Analyses (PRISMA) guidelines. The methodological rigor that we tried to follow allowed us to provide an overview of the use of the restorative approach and practices in schools and, at the same time, highlight its benefits. Restorative justice and restorative practices, as well as the restorative approach as a whole school-oriented disciplinary approach, provides a framework for prevention and intervention with respect to different aspects of school community life. The scientific literature on the subject underlines the importance of regularly evaluating the context and the availability of resources to strategically implement restorative justice programs and interventions in schools, especially in cases where the goal is to promote the restorative approach to the whole school as an alternative to traditional disciplinary policies and/or zero tolerance. It is essential to train all members of the school in restorative justice and practices, as well as a common and shared line among all members of the school community, also including families and external stakeholders who in various capacities work and/or collaborate with schools.

Although this review indicates positive outcomes for schools, teachers, students, and the school community at large, the criteria for the realization and implementation of the restorative approach and restorative practices in schools are changeable. For this reason, a systematic examination would allow, on the one hand, understanding more clearly the impact of restorative justice and practices in favouring the expected outcomes and, on the other hand, supporting schools in selecting and implementing effective projects and interventions to the objectives that they set themselves to ensure a positive, safe, respectful, fair, supportive, inclusive, well-being-oriented educational context. Furthermore, a qualitative summary, and not a meta-analysis summary, was performed in this review. Therefore, it should be emphasized that since this is a review of the literature, a systematic review including meta-analyses would certainly be necessary: this would allow detecting an exhaustive systematic search framework of the available evidence on the benefits and effectiveness of using RJ and PR at every level of school education.

Finally, further research in the field could focus on the specific challenges that school communities are experiencing nowadays. The COVID-19 pandemic has increased polarisation and hate in our communities, schools included, where conflicts arise between young people, teachers, parents, administrative staff. With such a common experience across schools, further research could focus on samples of schools adopting traditional methods or the restorative approach in their school culture to evaluate the potential differences in the way these community members react to and experience the situation.

## Appendix A

**Table A1:** Academic scientific literature excluded from the systematic review (*n* = 15).

First Author, Year	Study Design	Population	Intervention	Comparison	Outcomes
[60]	Quantitative research review (regression analyses, nonparametric models, tests, and analyses of variance) and literature review	US K–12 schools	Restorative justice and restorative practices as an alternative school disciplinary model	traditional school system	Reduction in misconduct (e.g., bullying); less use of exclusionary disciplinary responses (e.g., suspensions, expulsions); less unequal disciplinary treatment; higher school attendance; improvement of the school climate and safety; better academic results
[65]	Theoretical article	School	Restorative justice practices as an instrument of social development	restorative justice practices as an instrument of behavior management practice	Reflections on social development through restorative justice in New Zealand schools
[37]	Theoretical article	School	Restorative justice as a school reform strategy	punitive, exclusionary, and zero-tolerance approaches (e.g., suspensions, expulsions)	Reflections on exclusionary school discipline (ESD) as a question of health justice and on the importance of implementing school restorative justice practices to address ESD
[67]	Systematic literature review of peer-reviewed studies	Elementary, middle and high school students and teachers	Restorative conversations, circles, conferences, and peer mediation	exclusionary and punitive disciplinary policies (i.e., zero-tolerance policies)	Improved social skills (e.g., empathy, awareness, and accountability); prosocial behavior; positive school climate and social relationships between teachers and students and peers; problem-solving strategies; conflict management; reduction in bullying
[68]	Literature review	Elementary, middle, and high schools	Use of picture books to support social and emotional learning via RJ conversations and practices	traditional school system and practices	Improved social skills (e.g., empathy, awareness, and accountability); improved emotional skills, attitudes, academic performance; prosocial and positive behavior and building of safe and engaging classroom communities; positive school climate and social relationships; conflict and behavior management; boosted gender equality and understanding of different individuals and cultures
[69]	Theoretical article	School	Means of implementing models of positive discipline to counter racism and foster intercultural understanding	not available	The current literature on positive discipline fails to critically discuss issues of race and racism while calling for actions that reduce disciplinary disparity between students of different races; discussion of the implications and proposal of new criteria for policy, practice, and evaluation of positive discipline
[59]	Literature review	School	Implementation of a multilevel model of professional development to build teachers’ competences with respect to restorative justice and practices	traditional school system	Need for more research evaluating the impact of RJ in schools; promotion of alternative and multilevel methods of managing behavioral problems; improved social and emotional skills (e.g., empathy, awareness, and accountability); promotion of multilevel teacher training on RJ; promotion of school counseling for collaborative problem solving; development of a model of professional development of teachers to promote the implementation of RJ in school
[61]	Theoretical article	School	Analysis and comparison between restorative practices (RP), positive behavioral intervention and support (PBIS), and collaborative and proactive solutions (CPS) to manage behavior in the classroom and promote social justice	exclusionary and punitive disciplinary policies (i.e., zero-tolerance policies)	Collaborative approaches to problem solving; reduction in or elimination of traditional punishments; reduction in school exclusion and inequality based on gender, race, and socioeconomic status; improved social and emotional skills (e.g., empathy, awareness, and accountability); promotion of equity; prosocial and positive behavior; positive social relationships; restorative conferences to address serious behavioral incidents; lack of RJ training and preparation of teachers; most used RP in the UK; RP take time to be incorporated into the school system compared to the other approaches
[57]	Literature review	1378 primary and secondary schools in England	Effectiveness of restorative practices in dealing with cases of school bullying	“traditional” disciplinary practices oriented towards punitive approaches	Absence of coercion; proactive high-level conflict management; active involvement in the process of solving bully/victim problems
[70]	Theoretical article	School	RJ within an ecological framework	not available	Future directions for research, training, and practice
[71]	Systematic literature review	School	Restorative justice to promote anti-racism and social justice	not available	Importance of training for all school staff; importance of the support of external RJ professionals; promotion of a restorative peer culture to promote diversity and inclusion and prevent bullying; restorative practices in the classroom to create planned and joint actions to deal with, manage, and respond to conflicts and incidents; RJ schools can integrate into the approach racial justice education through the integration of specific contents and strategies; RJ to promote positive relationships, empowerment, and collaboration; RJ as an important predictor of improving school–family collaboration (even though there is still a lack of research); RJ allows schools to make changes in the whole system of thought, supporting the development of a fair and respectful school culture
[58]	Literature review	Students	Restorative approach and restorative practices (e.g., restorative conferences) as a disciplinary practice in school	exclusionary disciplinary policies (i.e., zero-tolerance policies)	Inclusive culture and reduction in inequality; self-awareness; development of skills for peaceful conflict resolution; improvement of positive relationships between peers and between peers and teachers; reduction in accidents, disciplinary postponements, risky behaviors, and school crimes; reduction in the need for extracurricular support
[49]	Theoretical article	School	Reflection on the critical theory applied to restorative practices in education	not available	Reflection on the current understanding, implementation, and sustainability of RJ practice in schools and more generally in education; the importance of understanding the restorative approach as an important factor of cultural transformation in the school and not just as practices for dealing with and managing conflicts and harmful behaviors
[73]	Theoretical article	School	Review of the current literature on restorative practices as an alternative approach to managing behavioral problems	traditional school system and approach	Safer schools; greater school connection; lower rates of suspensions and expulsions; work of psychologists fundamental for the theoretical, practical, and formative development as well as for the evaluation of RJ; inclusive culture and reduction in inequality; promotion of communication; improvement of positive relationships between peers and between peers and teachers; community-building circles to foster trust and develop bonds; reactive circles to tackle harm; restorative conferences to deal with serious accidents; improved social and emotional skills (e.g., empathy, awareness, accountability, responsibility)
[74]	Systematic review	34 studies	Restorative practices to reduce conflicts at school	traditional approach	Positive, safe, inclusive, and equitable school climate; development of social skills (e.g., empathy, awareness, responsibility, accountability); positive relationship between teachers and students and peers; reduction in health risk behaviors (smoking; substance use; dangerous sexual intercourse); decrease in suspension rates; lower likelihood of receiving disciplinary sanctions

## Appendix B

**Table A2:** Characteristics of the included studies (*n* = 34).

First Author, Year	Study Design	Population	Intervention	Comparison	Outcomes
[23]	Randomized controlled trial (RCT)	2771 students at 13 middle schools	Restorative practices to promote positive youth development paths and counter bullying and cyberbullying	traditional practices	Improvement of the positive school climate; peer attachment; social skills; reduction in victimization of bullying and cyberbullying; more positive behaviors
[29]	Follow-up survey	335 children from 32 public and private schools in Canberra, Australia	Restorative justice program to promote shame management skills and prevent school bullying	traditional behavioral problem management strategies (exclusionary and punitive disciplinary policies)	Increased shame management skills; development of reflective thinking; effective shame management responses; development of alternative conflict resolution practices
[30]	Correlational study	9921 students from 180 Denver public schools	Restorative interventions as a response and intervention for disciplinary problems at school	traditional disciplinary practices oriented towards punitive and exclusionary approaches	Highlighting of disciplinary disparities; significant association between participation in restorative justice interventions and positive results of the discipline; reduction in disciplinary sanctions
[24]	Randomized controlled trial (RCT)	students, school teachers and teaching assistants from 40 English secondary schools	Restorative approach implemented throughout the school to counter and manage incorrect behavior (e.g., bullying, aggression) through the formation of school action groups and external facilitation, staff training, and promotion of a new curriculum on social and emotional skills	normal practice	Reduction in bullying and aggression; reduction in health risk outcomes (e.g., substance use); promotion of mental health, emotional well-being, and QoL; reduction in NHS and social costs (e.g., costs of the justice system); increased access to restorative training; improved school environment; new opportunities for learning; more self-efficacy
[25]	Updates to the original trial protocol [24]	students, school teachers and teaching assistants from 40 English secondary schools	Restorative approach implemented throughout the school to counter and manage incorrect behavior (e.g., bullying, aggression) through the formation of school action groups and external facilitation, staff training, and promotion of a new curriculum on social and emotional skills	normal practice	Reduction in bullying and aggression; reduction in healthrisk outcomes (e.g., substance use); promotion of mental health, emotional well-being, and QoL; reduction in NHS and social costs (e.g., costs of the justice system); increased access to restorative training; improved school environment; new opportunities for learning; more self-efficacy
[26]	Randomized controlled trial (RCT)	students, school teachers and teaching assistants from 40 English secondary schools	Whole-school restorative approach to address bullying and aggression, involving school action group formation and external facilitation; staff training in restorative practices; a new social and emotional skills curriculum	normal practice	Improved social and emotional skills (e.g., empathy, awareness, and accountability); less bullying and aggression; reduction in student reports of bullying victimization; reduction in health risk behaviors (smoking; substance use, such as alcohol and drugs; dangerous sexual intercourse); reduction in the use of NHS services; better quality of life; greater emotional well-being; lower psychological difficulties
[35]	Single-case study	principal of one urban elementary school with 800 students	Restorative practices as a disciplinary model and practices in school	exclusionary and punitive disciplinary policies	Underestimation of the complexity of implementing restorative justice in schools with members of different races; need to evaluate the contextual characteristics of the school/community; importance of principal leadership in creating safe learning communities and school–community–family ties; importance of working together with teachers and families
[36]	Single-case study	principal of one high school with 1400 students	Restorative justice and restorative practices as an alternative school disciplinary strategy	traditional behavioral problem management strategies (exclusionary and punitive disciplinary policies)	Difficulty and complexity in implementing restorative justice; importance of creating an action plan shared by the entire school; importance of training on RJ; reduction in accidents, disciplinary postponements, and school crimes; importance of context analysis
[37]	Single-case study design	200 students from one urban high school	Inclusion of restorative approaches into the academic curriculum	traditional authoritarian and punitive school system	Development of non-hierarchical leadership; promotion of participatory and cocreative decision-making processes between the students and the school staff; improvement of positive relationships; greater sense of membership; human agency and resilience; promotion of positive social and emotional skills
[38]	Qualitative study (surveys, questionnaires, school data analysis)	412 students from 29 high schools	Restorative practices to promote positive relationships between teachers and students of all racial and ethnic groups	traditional school system	More equitable disciplinary practices; supportive and fair school climate; better student experience in the classroom and at school; greater respect perceived by students than their teachers; less use of disciplinary referrals for misconduct between racial and ethnic groups; positive relationships between teachers and students and peers
[39]	Qualitative research design (interviews)	18 school-based RP practitioners and principals from 13 schools in an urban district (three high schools, two combined schools, five middle schools, three elementary schools)	Restorative practices to enhance the skills of school staff and students and support for multilevel restorative practices	traditional school system	Identification of 12 indicators of restorative practices implementation; integration of an RP mindset into all aspects of the school; supportive and fair school climate; administrative support as a key to advancing schoolwide reform and restorative initiatives; development of alternative methods to the exclusionary discipline and conflict management; capacity building of school staff, students, and families; promotion of equity and social justice; inclusion of family and community members in school transition to PR
[27]	Randomized controlled trial (RCT) (interviews, questionnaires, triangulation of data)	12 middle schools	Restorative practices (circles, restorative conversation, restorative mediation, restorative conferences) as an alternative disciplinary practice in school	exclusionary and punitive disciplinary policies (i.e., zero tolerance-policies)	Reduction in expulsions, suspensions, truancy, and bullying; promotion of a sense of safety at school; increased teacher support; reduction in racial and socioeconomic disparities
[40]	Qualitative case study	one adolescent from one high school	Restorative justice and practices to decrease racial disparities in zero-tolerance school suspensions	exclusionary and punitive disciplinary policies (i.e., zero-tolerance policies)	Future of presentations, increased sense of academic value and community support; development of the ability to recognize the experience and reality of marginalized student groups
[31]	Interrupted time series (ITS) analysis	Los Angeles Unified School District (about 100 middle and high schools)	Suspension bans and restorative justice programs to lower student suspension rates	exclusionary and punitive disciplinary policies	Alternative conflict resolution practices; lower withdrawal rates; reduction in unequal treatment
[41]	Qualitative case study (focus groups, interviews, open-ended survey questions, triangulation of data)	teachers, students, families, university students, and community of one elementary school	Restorative justice approach and practices as a disciplinary model in school	traditional school system	Development of a peer mediation program; positive classroom and school relationship and climates; reduction in violent behavior; development of conflict resolution strategies; reduction in social exclusion and diversity; greater scholastic and academic involvement; increased commitment from parents and the community; improved social skills (e.g., empathy, awareness, and accountability)
[42]	Qualitative study (interviews, semi-structured interviews, focus groups)	40 students, 14 teachers, six principals from six elementary, middle, and high schools	Implementation of the restorative justice approach and practices as a disciplinary model in school to promote behaviors, relationships, and a positive school climate	traditional school system	Improved social skills (e.g., empathy, awareness, and accountability); reflective thinking; reduction in behavioral problems (aggression, bullying) and greater management skills; positive school climate and social relationships between teachers and students and peers; reduced rates of anxiety and depression
[43]	Qualitative case study	17 teachers and staff members and two parents from one elementary school	Year one implementation of the restorative justice approach and practices	exclusionary and punitive disciplinary policies	More positive behaviors; building of safe and engaging school classes; positive school climate and social relationships between teachers and students, families and peers; improved social skills (e.g., empathy, awareness, and accountability)
[44]	Qualitative study (interviews, observations)	Students and teachers at three small public schools in an urban district	Collection of the teachers’ experiences in implementing community-building circles to promote equity and inclusion	traditional school system	Difficulty of teachers in fully implementing community-building circles due to lack of adequate training and networking with other professionals in the sector (e.g., psychologists, social workers); opportunities for participants to get to know each other, fostering respect for each other and for diversity; new opportunities to teach, learn, and practice; promoting of shared learning to build mutual trust and growth; promotion of the ability to share emotions
[32]	Nonexperimental design (follow-up immediately post-training and six months later; recall session discussions; multiagency focus groups)	50 primary schools and nine secondary North Ayrshire Council schools (Scotland)	Effectiveness of an educational psychology service in the implementation of activities with the restorative approach	Not available	Increased use of restorative practices/activities; importance of collaboration with out-of-authority personnel to offer quality training; great impact of school leadership on the levels of the RA implementation at individual schools achieved; importance of collegiate support within an organization, school’s ethos, and readiness for change; importance of EPS representation in school partnership to ensure its consistency with the RA
[33]	Nonexperimental design (T1 and T2 evaluation, implementation and delivery of restorative programs in three schools through the efforts of the local youth offending teams (YOTs))	three schools (middle and high schools)	Promotion of the restorative approach to promote well-being, in particular happiness and scholastic commitment	normal practice	Difficulty in drawing definitive conclusions on the impact of restorative practices on well-being, in particular on happiness and school commitment; evaluation of the impact of three different RA models; the RA could influence results in very specific contexts; potential of the restorative approach in positively influencing outcomes; higher levels of happiness and scholastic engagement in schools that had implemented the restorative approach to the whole school; lack of empirical evidence, especially regarding the positive influence of restorative programs on happiness: it is unclear which restorative practices are responsible for this positive influence
[45]	Practice-based qualitative research (interviews, observations)	four middle-grade public school teachers	Restorative dialogue practices (circle time and peace circles)	traditional hierarchical, authoritarian, and punitive school system	Building of an inclusive class community; greater communication skills through participation in dialogue; proactive conflict management; promotion of social skills (e.g., empathy, awareness and accountability)
[55]	Quantitative study (external data, school data analysis, student and teacher questionnaires)	294 public nonalternative secondary schools	Exploration of the effect of racial composition of the school on the use of punitive and/or restorative disciplinary responses	not available	Schools with a larger population of Black/Hispanic and low-income students are more likely to use punitive practices for management/responding to misbehavior; the use of severe punishment increases the likelihood of choosing punitive/zero-tolerance disciplinary policies; the use of milder punishments increases the likelihood of using restorative practices; schools with qualified managers and staff are more likely to use both punitive and restorative disciplinary responses
[52]	Literature review and qualitative research (questionnaires, semi-structured interviews)	three experienced experts in the field of peer mediation in Croatian schools (number of schools not available)	Nonviolent conflict resolution and implementation of peer mediation in school settings	traditional behavioral problem management strategies (e.g., exclusionary and punitive discipline)	Improved social and emotional skills (e.g., empathy, awareness, and accountability); development of reflective thinking; development of nonviolent conflict resolution strategies; problem-solving strategies; increase in positive social relationships between peers
[46]	Qualitative case study (document analysis, participant observation, interviews, educator questionnaires, and learning circles)	one Canadian primary school	Role of restorative justice in facilitating student well-being	traditional approach	Greater sense of individual and collective coherence; greater ability to reflect on and understand daily existence; greater ability to offer support to others; greater ability to manage behavioral problems and conflicts; participation and involvement
[47]	Two qualitative case studies (interviews and informal check-ins)	two restorative justice coordinators at two high schools	RJ interventions to address racial disparity	traditional approach	Development of formal and informal networks; development of racial reflexivity and critical conversations about race, highlighting that the Whites and the Blacks differ in perception of racial discrimination; improved relationships between the Whites and the Blacks
[48]	Qualitative case studies (focus groups, interviews, semi-structured observations)	five middle and high schools	Restorative justice approach and restorative practices (e.g., restorative conversations, circles, restorative conferences, peer mediation) to build a restorative school community	traditional hierarchical, authoritarian, and punitive school system	Inclusive culture and reduction in inequality; positive school climate and relationships between peers and between peers and teachers; better school–family relationships; proactive conflict management; reduction in accidents, disciplinary postponements, and risky behaviors
[53]	Qualitative and quantitative secondary survey	90 students from elementary and middle schools (number of schools not available)	Proactive implementation of restorative practices and exploration of student perceptions of the impact of restorative circles on behavior and conflict	“traditional” disciplinary practices oriented towards punitive and exclusionary approaches	Promotion of communication; higher capacity to express thoughts and feelings; improved social skills (e.g., perspective-taking, awareness, and accountability); promotion of nonviolent problem-solving strategies; improvement of positive relationships between peers and between peers and teachers
[56]	Quantitative study using the 2013/2014 CHKS and a part of the CALSCHLS system (surveys targeting students, parents, and school staff)	6992 students from 32 middle and high schools in one California school district	Effects of school-based restorative justice on physical health, mental health, and academic achievement	traditional school system	Inability to evaluate the cause and effect due to the crosscutting nature of the data; limited generalizability; importance of assessing the association between restorative justice and health impact, depression, and self-reported behavior; reduction in cases of absence for health reasons; better academic performance; higher school attendance rates; whole-school restorative approach can be a significant predictor in several respects; peer restorative justice is a significant predictor of health-related positive outcomes; need for further qualitative and quantitative studies
[49]	Qualitative case study design (on-site observation, policy analysis, and semi-structured interviews)	12–15 educators in two schools in Ontario	Exploration of ways to implement restorative justice in school education settings to promote relational and peaceful school culture	normal practice	Little attention to the structural elements of the school; difficulty in dealing with structural and institutional influences on the school community and RJ; little awareness of the importance of the professional contribution of the whole school to changing its culture and system; importance of providing professional tools for more effective implementation of alternative educational practices
[54]	Multiple-case study (qualitative and quantitative measurements and data collection methods)	13 teachers and 40 staff members from four urban schools (two elementary schools, one middle school, and one high school)	Restorative circles to improve school climate and promote alternative behavioral problem management, peaceful conflict resolution, and community building	not available	Reactive circles to deal with behavioral problems/incidents and repair relationships; positive impact of reactive circles on students’ attitudes and behaviors; reduction in punitive measures; need for training and support to support teachers in the use of restorative circles
[28]	Randomized controlled trial (RCT) (interviews, focus groups, observations, and questionnaires)	students and teachers from secondary schools (number of schools not available)	Action groups (AG) made up of students and teachers trained in restorative justice and practices (in particular, restorative conferences, and circle time) and external facilitators	non-use of action groups trained in restorative justice	Fewer experiences of violence and bullying; greater well-being and better quality of life; reduction in health risk behaviors (smoking; substance use; dangerous sexual intercourse); reduction in the use of NHS services; more students engaged in education (reduction in absenteeism); greater inclusion; improved communication and relationships between peers and staff and students
[50]	Qualitative case study design (interviews, observations, and review of documents)	students, teachers, and an administrator from one middle school	Restorative approach and restorative practices (e.g,. respect agreement, letter-writing process) as a disciplinary practice in school	traditional disciplinary practices oriented towards punitive approaches	Improved social skills (e.g., empathy, awareness, and accountability); autonomy; reflective thinking; positive relationship with teachers and peers; equitable, safe, supportive, inclusive environment; greater adherence to the rules; higher levels of academic achievement; decrease in absenteeism
[51]	Qualitative case study design (focus groups, observations of classrooms and school board meetings, conversations, sets of ethnographic notes, institutional documents and data)	students, teachers, families, and school administrators from two middle schools and one high school	Restorative justice to decrease racial disparities in zero-tolerance school disciplinary measures	exclusionary and punitive disciplinary policies (i.e., zero-tolerance policies)	Development of a restorative justice program; adoption of a code of conduct for restorative practices; greater understanding and discussion of the causes of conflicts and transgressions; reduction in suspension rates; development of social skills (e.g., empathy, awareness, responsibility, accountability)
[34]	Quasiexperimental pre–post design	1480 high school students from four different Hong Kong schools	Restorative approach to the whole school to counter and manage bullying	traditional approach	Development of inclusive and respectful culture; reduction in bullying; higher social skills (e.g., empathy, awareness, and accountability); higher self-esteem; development of a positive school climate; importance of training for all members of school staff; conflict resolution strategies
